# DNA Methylation Patterning and the Regulation of Beta Cell Homeostasis

**DOI:** 10.3389/fendo.2021.651258

**Published:** 2021-05-07

**Authors:** Nazia Parveen, Sangeeta Dhawan

**Affiliations:** Department of Translational Research and Cellular Therapeutics, Arthur Riggs Diabetes and Metabolism Research Institute, City of Hope, Duarte, CA, United States

**Keywords:** diabetes, beta cells, insulin, DNA methylation, epigenetics

## Abstract

Pancreatic beta cells play a central role in regulating glucose homeostasis by secreting the hormone insulin. Failure of beta cells due to reduced function and mass and the resulting insulin insufficiency can drive the dysregulation of glycemic control, causing diabetes. Epigenetic regulation by DNA methylation is central to shaping the gene expression patterns that define the fully functional beta cell phenotype and regulate beta cell growth. Establishment of stage-specific DNA methylation guides beta cell differentiation during fetal development, while faithful restoration of these signatures during DNA replication ensures the maintenance of beta cell identity and function in postnatal life. Lineage-specific transcription factor networks interact with methylated DNA at specific genomic regions to enhance the regulatory specificity and ensure the stability of gene expression patterns. Recent genome-wide DNA methylation profiling studies comparing islets from diabetic and non-diabetic human subjects demonstrate the perturbation of beta cell DNA methylation patterns, corresponding to the dysregulation of gene expression associated with mature beta cell state in diabetes. This article will discuss the molecular underpinnings of shaping the islet DNA methylation landscape, its mechanistic role in the specification and maintenance of the functional beta cell phenotype, and its dysregulation in diabetes. We will also review recent advances in utilizing beta cell specific DNA methylation patterns for the development of biomarkers for diabetes, and targeting DNA methylation to develop translational approaches for supplementing the functional beta cell mass deficit in diabetes.

## Introduction

All of the distinct cell types in a multicellular organism share the same DNA sequence, and yet the phenotype of each individual cell type is unique, and dictated by the cell-type specific patterns of gene expression. In addition, an individual cell type can often adapt its behavior in response to changing environment by modifying its gene expression. This context specific interpretation of the genome is facilitated through a set of mechanisms that impact the accessibility of genomic regions. These mechanisms include covalent modifications of DNA and DNA binding proteins such as histones, as well as RNA species such as microRNAs and long non coding RNAs, with DNA methylation being the most well-known direct modification of the genome (1). Collectively, these mechanisms are referred to as epigenetic mechanisms. The term ‘*epigenetics*’ was first used by Waddington in 1942 to describe the influence exerted by the environment on the genome towards shaping the cellular phenotype during development (2). More recently, Arthur Riggs gave a mechanistic dimension to this term, and defined epigenetics as “the study of mitotically and/or meiotically heritable changes in gene function that cannot be explained by changes in DNA sequence” (3). In its present form, this term has come to encompass all transient or stable changes in chromatin structure that mark or perpetuate altered activity, in response to an environmental cue (4). Epigenetic changes permit a cell to alter its gene expression and phenotype in response to any changes in the environment, in contexts such as generating cellular diversity in response to morphogens during development, or adaptation of cellular behavior to changing nutrient availability or exposure to stress stimuli.

DNA methylation is one of the earliest and most well studied epigenetic modifications (5, 6). Methylation of DNA occurs predominantly at the cytosine residue within CpG dinucleotides in mammalian cells and serves to regulate gene expression (7, 8). DNA methylation levels at repetitive elements and CpG dense regions around promoters do not vary much between different cell types. However, the patterns of DNA methylation at regions that regulate gene expression, such as enhancers, are very cell type specific and essential for the maintenance of cellular phenotype [reviewed in (9)]. This suggests that any changes in DNA methylation patterns can potentially impact cell-fate and function. Depending on the stimuli triggering such changes and the genes impacted by them, altered DNA methylation patterns can either facilitate adaptation to the changing environment, or disrupt cellular function and lead to pathological changes. This view is supported by the plastic nature of DNA methylation and the existence of enzymatic mechanisms that can erase DNA methylation marks (10). A large number of studies have demonstrated that stage specific patterning of DNA methylation signatures is essential for cell-fate specification and functional maturation of pancreatic beta cells, the major cell type that regulates glucose homeostasis [reviewed in (9, 11)].

Beta cell failure plays a central role in the pathogenesis of diabetes, a disease that impairs the body’s ability to regulate blood glucose. Accumulating evidence suggests that loss of beta cell identity and functional maturity contribute to beta cell dysfunction in diabetes (12). Comparison of genome-wide DNA methylation profiles in islets from diabetic and non-diabetic subjects reveals that DNA methylation patterns related to beta cell function and identity are altered in diabetic islets (13). The formation and erasure of DNA methylation patterns is tightly linked to cellular metabolism. It is therefore likely that an altered metabolic milieu in diabetes drives epigenetic disruptions underlying beta cell failure. The influence of environmental factors such as diet and exercise on metabolic health and diabetes risk is now well recognized, and further underscores the importance of epigenetic regulation in glucose homeostasis (14, 15). In this review, we focus on the essential role of DNA methylation in the establishment and maintenance of the functional beta cell phenotype, and the relevance of altered DNA methylation patterns to diabetes pathophysiology. We will also discuss emerging approaches that aim to harness beta cell specific DNA methylation signatures as biomarkers for diabetes, and highlight proof-of-principle studies that demonstrate the therapeutic potential of targeting DNA methylation to promote beta cell function and regeneration.

## DNA Methylation and Beta Cell Homeostasis

### DNA Methylation: A Key Epigenetic Module

**Figure 1:**
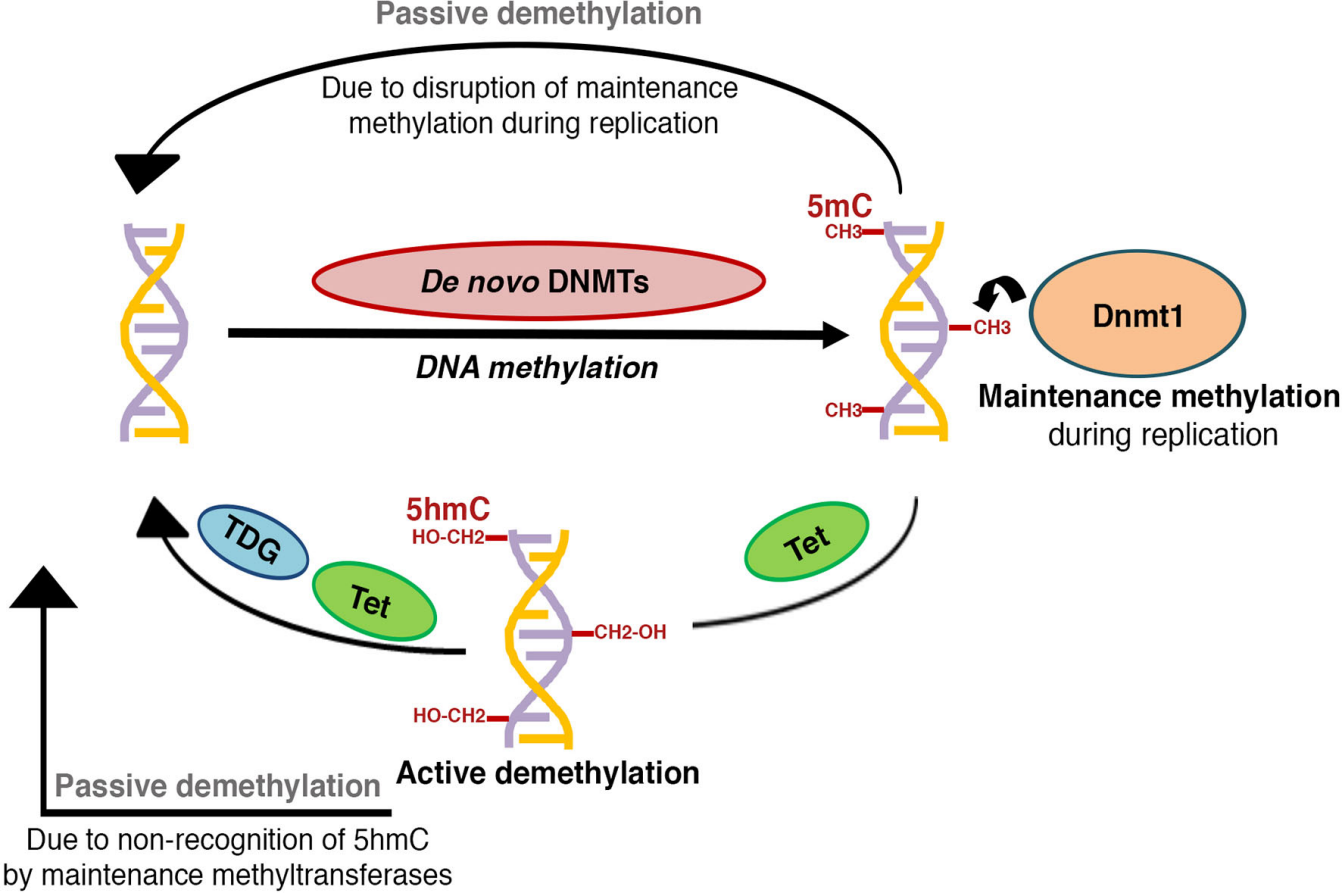
Schematic representation of DNA methylation patterning: The establishment of new DNA methylation patterns during development is regulated by the activity of *de novo* DNA methyltransferases, while activity of maintenance DNA methyltransferases serves to perpetuate these patterns during successive rounds of cell division. DNA methylation marks can be reversed through active or passive demethylation. Active demethylation involves the successive enzymatic oxidation of 5-methylcytosine (5mC) to 5-hydroxymethylcytosine (5hmC),5-formylcytosine (5fC), and 5-carboxylcytosine (5caC) by TET (Ten-eleven translocation) dioxygenases, followed by thymine DNA glycosylase (TDG) dependent removal of 5fC and 5caC, coupled with base-excision repair to a cytosine (C). A hemi-methylated 5hmC is not recognized by the maintenance DNA methyltransferases and can get diluted and lost during replication, thus contributing to passive demethylation. Disruption of maintenance methyltransferase activity can similarly result in replication dependent dilution of DNA methylation.

DNA methylation is a heritable epigenetic modification that involves the covalent addition of a methyl group to the bases, most predominantly at the 5th carbon of a cytosine within the CpG dinucleotides, leading to the formation of 5-methylcytosine (5mC) (16). 5mC is the most well characterized and abundant direct modification of DNA in the mammalian genome. In contrast, methylation of non-CpG sites occurs at a very low frequency (17), and is beyond the scope of this review. The addition of methyl group on cytosine is carried out by a family of enzymes called DNA methyltransferases (Dnmts), that includes Dnmt1, Dnmt2, Dnmt3a, Dnmt3b and Dnmt3l [reviewed in (18, 19)]. Dnmt1, Dnmt3a, and Dnmt3b are the *bona fide* DNA methyltransferases that have the catalytic activity, while Dnmt2 and Dnmt3l lack DNA methyltransferase activity of their own, and are likely to play an allosteric regulatory role (19). DNA methylation has two distinct regulatory layers: *de novo* methylation, or the formation of new methylation patterns (20–22), and maintenance methylation, or the post-replication restoration of existing methylation patterns on the nascent strand (22–24) (**Figure 1**). *De novo* methylation is primarily carried by Dnmt3a and Dnmt3b (21), while Dnmt1, the first methyltransferase to be discovered, serves to maintain methylation patterns through replication and prefers hemi-methylated CpG sites (23, 25).

DNA methylation plays a central role in the regulation of gene expression and maintenance of genomic integrity, and directs processes such as gene repression, silencing of transposons, genomic imprinting, and X- chromosome inactivation [reviewed in (7, 8, 26, 27)]. It is noteworthy that the target of DNA methylation regulation, the CpG dinucleotides, represent only about 1% of the mammalian genome. A large proportion of CpG residues are clustered in CpG-rich regions called the CpG islands (CGIs), which are typically devoid of methylation [reviewed in (28)]. About half of the CGIs are located proximal to the transcription start sites (TSSs), and need to remain hypomethylated for promoter activity (29). While the hypomethylation of promoter-associated CGIs is common to most cell types, the DNA methylation patterns at enhancer are cell type specific and indicate the status of enhancer activity (9). In contrast to the association of promoter CGI methylation with gene repression, gene body methylation typically indicates active transcription [reviewed in (16)]. This suggests that DNA methylation can repress, permit, or modify gene expression, depending on the genomic context. The overall low frequency of CpG residues and observed paucity of CGI methylation has likely evolved to protect against mutational loss of CpGs due to 5mC deamination, while creating an in-built selectivity to DNA methylation dependent regulation. Transcriptional regulation by DNA methylation involves the recognition of methylated CpGs, residues by DNA methylation “readers” such as the “methyl-CpG binding domain” proteins or MBD proteins, which recruit chromatin regulatory enzymes that establish appropriate epigenetic modifications to create a specific regulatory chromatin milieu [reviewed in (8, 30)]. Additional specificity of DNA methylation is achieved by interaction of MBD complexes and/or methylated regions with stage-specific transcription factors, to regulate DNA methylation at their binding sites (31–33).

While it has long been recognized that DNA methylation is dynamic in nature, the mechanisms mediating the active removal of 5mC remained largely understudied until a decade ago (10, 34). 5mC marks can be reversed through two mechanisms: active demethylation and passive demethylation [reviewed in (10, 16, 34)] (**Figure 1**). Active demethylation of CpG residues involves the step-wise enzymatic oxidation of 5mC to 5-hydroxymethylcytosine (5hmC), 5-formylcytosine (5fC) and 5-carboxylcytosine (5caC) by TET (Ten-eleven translocation) dioxygenases (35, 36). 5fC and 5caC can then undergo base excision mediated by thymine DNA glycosylase (TDG) leading to active erasure of methyl marks [reviewed in (10)]. Alternative pathways, involving Growth Arrest and DNA Damage-inducible (GADD) 45 proteins (GADD45A and GADD45B), AID (activation induced cytidine deaminase) and APOBEC (apolipoprotein B mRNA editing enzyme, catalytic subunit) have been reported [reviewed in (10, 37)], but the extent of their contribution to DNA demethylation remains unclear. Passive demethylation, on the other hand, can result from dilution of 5mC during replication due to the failure of maintenance methylation, followed by dilution through subsequent rounds of replication [reviewed in (10)]. Collectively, both active and passive demethylation contribute to the homeostasis and remodeling of DNA methylation.

The establishment and maintenance of DNA methylation patterns is essential for embryonic development and differentiation. Early embryonic development in mammals is marked by two waves of global demethylation and re-methylation, the first, which occurs immediately after fertilization and constitutes the pre-implantation reprogramming, and the second, which happens in the primordial germ cells (PGCs) during gametogenesis [reviewed in (38–40). Such demethylation creates a clean epigenetic slate that underlies developmental multipotency, and can then be used to establish stage specific DNA methylation programs during differentiation. However, this also creates a particularly sensitive developmental window, during which any adverse environmental exposures can have a significant impact on cell phenotypes in later life. During embryonic development, the *de novo* methyltransferases establish the new methylation patterns in the inner cell mass (ICM) after implantation, and maintenance of these patterns by Dnmt1 ensures continuity of the epigenetic state through cell division (38–40). Lineage specific refinement of DNA methylation patterning of promoters and enhancers then guides the cell-type-specific gene-expression patterns throughout the differentiation program (reviewed in [9)]. Overall, *de novo* and maintenance methylation perform an indispensable role in shaping mammalian development through guiding the differentiation and growth of different organs, including pancreas.

### DNA Methylation in Pancreas Development and Beta Cell Specification

The pancreas is a multifunctional organ, performing both exocrine and endocrine functions, namely the secretion of digestive enzymes and hormones (41). The exocrine component of the pancreas comprises of acinar cells that synthesize digestive enzymes, and ductal epithelium that transports those enzymes into the gut. On the other hand, the endocrine pancreas consists of alpha- (α), beta- (β), gamma- (γ), delta- (δ), and epsilon- (ϵ) cells, which cluster together in the islets of Langerhans to regulate glucose homeostasis by secreting hormones-glucagon, insulin, pancreatic polypeptide, somatostatin, and ghrelin, respectively into the blood stream [reviewed in (42, 43)]. Pancreas is an endodermal lineage organ, and after gastrulation, a series of developmental steps lead to the formation of the primitive gut tube from endoderm (44). Pancreatic development begins with the emergence of dorsal and ventral anlagen that harbor multipotent progenitor cells (MPCs), on opposite sides of the foregut endoderm at embryonic day 9.0 (E9.0). Following the rotation of the gut tube, these buds fuse around E12.5 to form the full organ, with continued expansion and branching of the pancreatic epithelium, accompanied by the differentiation of pancreatic progenitors into endocrine, acinar, and ductal lineages [reviewed in (42–46)]. The differentiation of pancreatic progenitors into the endocrine lineage involves an intermediate, Neurogenin3 (Neurog3) expressing endocrine progenitor stage. By the end of gestation, the developing pancreas has acquired its typical structure, with acinar rosettes surrounding the ends of the ductal tree and clusters of endocrine cells scattered throughout the organ [reviewed in (41, 42)].

Dynamic regulation of DNA methylation plays an important role in the development of pancreas from definitive endoderm and further specification of endocrine cells. *De novo* methylation patterning plays a critical role in this stepwise differentiation process; with Dnmt3a being the primary *de novo* DNA methyltransferase in the endocrine lineage, while Dnmt3b is restricted to the acinar lineage (47). A recent study on the *in vitro* differentiation of human pluripotent stem cells (hPSCs) into pancreatic islets revealed that pluripotency genes undergo DNA hypermethylation to enable chromatin silencing, and that hypomethylation of lineage specific genes drives stage-specific chromatin activation during endocrine specification (48). The hyper- and hypo-methylation of genomic regions coincides with the repression or activation of the nearest gene, and enables stage-specific gene expression during different stages of endocrine differentiation (48). DNA methylation and demethylation patterns also play a central role in regulating the recruitment and activity of pioneer transcription factors (TFs), such as the forkhead box proteins FOXA1 and 2, which direct lineage specific chromatin remodeling and enhancer priming during endocrine differentiation (49–55). Genome-wide mapping of 5mC and 5hmC patterns during differentiation of human embryonic stem cells (hESCs) to pancreatic lineage shows that global 5hmC levels are reduced, with a corresponding increase in 5mC during the formation of definitive endoderm. However, 5hmC levels gradually increase during subsequent differentiation steps, with a concomitant 5mC decline (54, 56), similar to the differentiation of other progenitors (57). 5hmC enrichment also positively correlates with increased chromatin accessibility and lineage specific enhancer activity (54). In line with this, deletion of all three TET enzymes in hESCs leads to impaired differentiation of pancreatic endoderm, corresponding to the disruption of stage-specific 5hmC patterning (55). Thus, pioneer factors such as FOXA2 guide the establishment of lineage specific 5hmC patterns on specific genes, which in turn govern chromatin accessibility and maintenance of the epigenetic landscape in active genomic regions at a given developmental stage. This suggests that an enrichment of global 5hmC levels is a conserved hallmark of cell differentiation. In alignment with this, the differentiated alpha and beta cells appear to be globally hypomethylated compared to various preceding developmental stages during hPSC differentiation (48).

**Figure 2:**
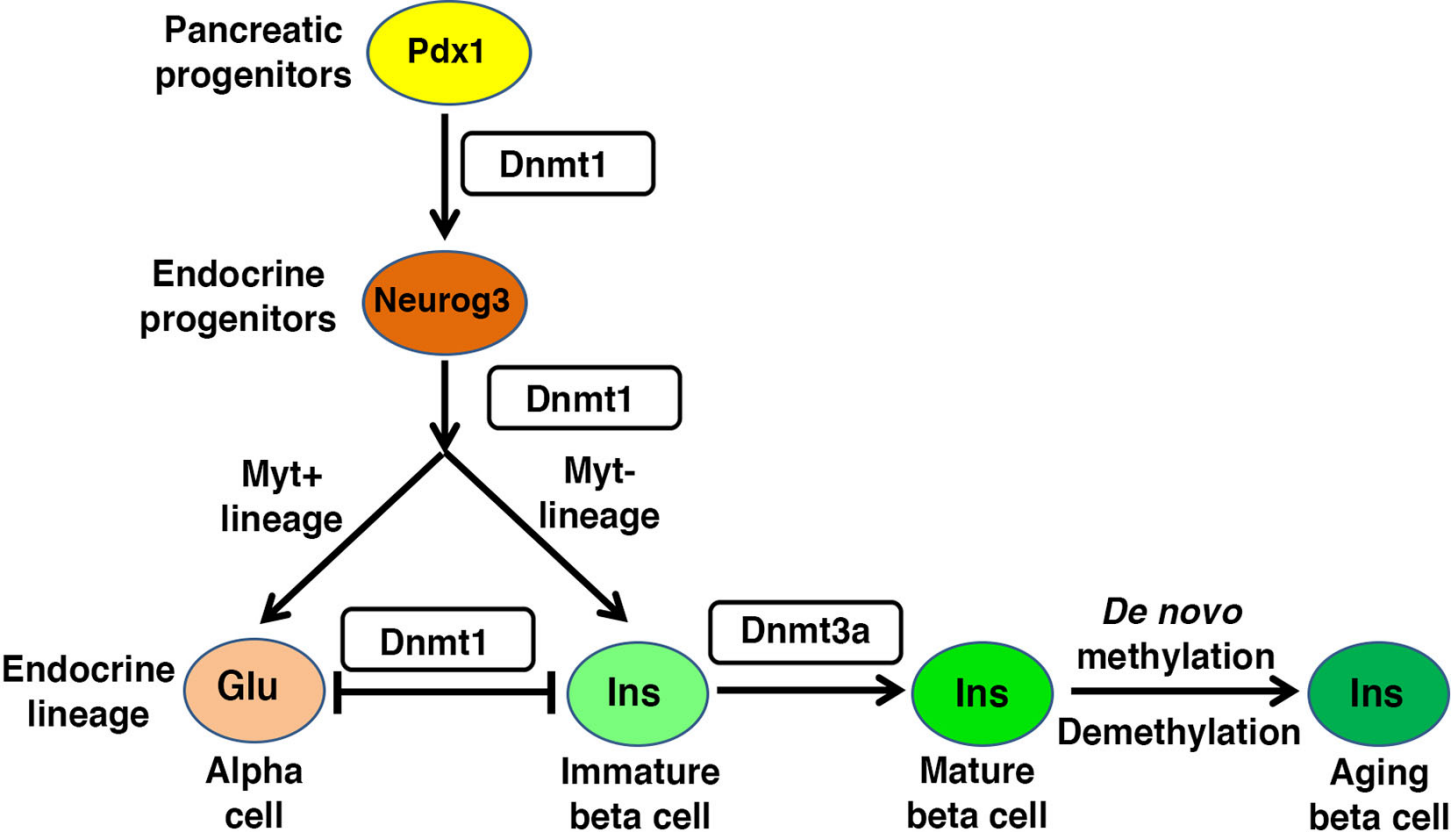
Regulatory role of DNA methylation in beta cell homeostasis: Pancreas morphogenesis requires intact maintenance methylation activity of Dnmt1 in Pdx1+ pancreatic progenitors, and loss of Dnmt1 in pancreatic progenitors leads to pancreatic atrophy. Specification of different endocrine lineages from the Neurog3+ endocrine progenitors involves the DNA methylation dependent regulation of the expression of lineage-specific transcription factors. The endocrine progenitors that co-express Myt1, are marked by high expression of Dnmt1 and hypermethylation of the enhancer region of *Arx*, a key alpha cell lineage determinant. This leads to repression of *Arx* and commitment of the Neurog3+ Myt1+ sub-population to beta cell lineage, while the Neurog3+ Myt1- sub-population acquires alpha cell lineage. Functional maturation of beta cells is neonatal life depends on DNA methylation patterning of genes involved in metabolism and replication. The fully differentiated beta cell phenotype is guarded by maintenance methylation through replication, and loss of Dnmt1 in beta cells leads to their trans-differentiation into alpha cells. The proliferative capacity and function of beta cell changes with age, and involves age dependent changes in the beta cell methylome.

Genetic manipulation studies have provided valuable insights into the role of DNA methylation in pancreatic endocrine development. Loss of the maintenance DNA methyltransferase, Dnmt1, in both zebrafish and mice, leads to defects in pancreas development (58, 59). Dnmt1 not only preserves epigenetic information in replicating cells, but also maintains genomic integrity *via* centromere methylation (60, 61), such that loss of Dnmt1 causes genomic instability during cell division and triggers p53 dependent cell-death. In mice, Dnmt1 is required to repress *p53* expression and promote progenitor survival during pancreatic progenitor differentiation. *Dnmt1* ablation in mouse pancreatic progenitors leads to activation of p53 dependent apoptosis that results in pancreatic atrophy, a defect that can be rescued by *Trp53* haploinsufficiency (59). This suggests that maintenance of DNA methylation signatures is important for the regulation of key checkpoints in the rapidly dividing pancreatic progenitors (**Figure 2**). Pancreatic progenitor give rise to endocrine cells *via* a Neurog3 expressing endocrine progenitor stage [reviewed in (41, 42)]. The differentiation of endocrine progenitors to specific islet cell-fates depends on differential DNA methylation of lineage restricted enhancers, with no apparent differences in promoter methylation between different endocrine cell types (62, 63). The promoter regions of insulin and glucagon genes are rapidly demethylated during endocrine progenitor differentiation, regardless of the specific destined endocrine lineage. Accordingly, promoters of genes encoding insulin and glucagon lack DNA methylation in alpha-, beta- and delta-cells, independent of their expression in these lineages. Instead, endocrine cells exhibit differential methylation in the enhancer regions of these genes, indicating that endocrine cell identity is regulated by enhancer methylation (62). Genome-wide comparison of human alpha- and beta-cell methylomes shows the conservation of this phenomenon in humans, such that lineage specific methylation differences are concentrated in enhancers (62). The shared promoter hypomethylation of lineage-specific genes across endocrine cell-types likely subserves the changes in endocrine identity that occurs in the context of beta cell regeneration and diabetes pathogenesis.

DNA methylation directs the specification of different endocrine cell-fates during early pancreatic development by modulating expression of key genes encoding cell-fate regulatory transcription factors (31, 63–65) (**Figure 2**). For instance, the specification of beta cell identity is regulated by the DNA methylation dependent repression of *Aristaless homeobox* (*Arx*) locus, which encodes a master regulator of alpha cell identity (31, 64). The DNA methylation dependent repression of *Arx* is established by the recruitment of a complex containing Dnmt3a with transcription factors Nkx2.2, Nkx6.1, Grg3 and histone deacetyalse-Hdac1 to the *Arx* promoter in beta cells (31). Beta cell lineage specification also requires the TET1 dependent hypomethylation at enhancer regions of beta cell specification genes (55), such as Pax4 which represses *Arx* expression in beta cells (66–68). This suggests that epigenetic regulation directs lineage specification in coordination with transcription factor recruitment. However, lineage specific factors such as Arx and Pax4 are co-expressed in nascent Neurog3+ endocrine progenitors (67, 68), suggesting the existence of mechanisms that restrict factors such as Arx and Pax4 to different lineages. Recent data suggests that the epigenetic roadmap for such restriction is established prior to endocrine progenitor specification. Pancreatic progenitors represent a transcriptionally homogenous pool before differentiating into the Neurog3+ lineage, but differ in DNA methylation pattern at the enhancers of key cell-identity genes, which primes them towards distinct endocrine lineage programs. This epigenetic heterogeneity is regulated by Myt1, such that Neurog3^+^ cells that co-express Myt1 are marked by higher Dnmt1 levels and hypermethylation of *Arx*, which biases them to a beta cell-fate (63) (**Figure 2**). These data point to a surprising role for maintenance methylation in directing the differentiation of endocrine progenitors. Besides Dnmt1, epigenetic regulators such as arginine methyltransferase Prmt5, histone deacetylase Hdac3, and lysine demethylase Kdm4a are differentially partitioned between the two endocrine progenitor sub-populations (63), suggesting that DNA methylation patterning works in conjunction with other epigenetic modules to direct endocrine cell-fate choices. However, the spatio-temporal and signaling events that guide such epigenetic heterogeneity, and the relative roles of *de novo* versus maintenance methylation in this process remain to be elucidated. Emerging advances in single cell epigenome profiling methods will be useful in clarifying some of these issues. Overall, endocrine differentiation of cells involves the dynamic and highly stage specific patterning of 5mC and 5hmC marks, which is coordinated by an interplay of stage specific activities of pioneer factors, lineage determinant transcription factors, DNA methyltransferases, and DNA demethylases. This not only determines the sequence specificity of DNA methylation, but also guides the epigenetic priming of lineage specific regulatory regions to orchestrate the stage-specific developmental program in response to instructive signals during pancreatic differentiation.

### DNA Methylation in Beta Cell Maturation and the Maintenance of Full Functional Beta Cell Phenotype

Beta cell mass expands significantly by replication in the late fetal to early postnatal life corresponding to the high rates of growth, in both mice and humans (69–73). However, beta cells during this growth phase are functionally immature and display a lower threshold of glucose concentration for insulin secretion. Accordingly, they secrete insulin secretion even at low glucose levels and are therefore not glucose responsive (47, 74, 75). Immature beta cells utilize anaerobic glycolysis and higher amino acid metabolism (76–79), which allows a low glucose threshold and supports the high rates of replication in this phase (47, 77, 79). Beta cells acquire glucose sensitivity as they exit cell-cycle, suggesting an inverse correlation between beta cell replication and functional maturity (80). In mice, the process of functional maturation begins around postnatal day 7 and is complete at weaning (75, 81, 82), while in humans, beta cell maturation occurs within the first 2-3 years of life (73, 83). This transition from a replicative, glucose non-responsive to a quiescent, glucose-responsive state is coupled with a metabolic changes that allow beta cells to establish a higher threshold for glucose, and amplify ATP production through oxidative phosphorylation (84). This metabolic switch from anaerobic- to aerobic-glycolysis is directed by the *de novo* DNA methyltransferase Dnmt3a. Methylation at distal promoter regions of the so-called “disallowed” genes, such as the low Km hexokinases-1 and -2 (*Hk1*, *Hk2*) and lactate dehydrogenase A (*Ldha*), leads to their repression and allows the coupling of insulin secretion to glucose levels (47). Ablation of Dnmt3a in beta cells leads to higher basal insulin secretion even in adult life, similar to neonatal beta cells, and renders them functionally immature. This process is in part controlled by changes in the levels of Dnmt and Tet enzymes; such that Dnmt3a and Tet levels decline after maturation (47, 53). Thus, establishment of the functional beta cell phenotype is dependent upon the DNA methylation mediated repression of glucose-secretion decoupling genes (47).

The changes in beta cell metabolic program during maturation coincide with and reflect a change in nutrient quality and accessibility (85). Studies in mice have shown that weaning induces a distinct, final step of functional maturation that is coupled with a dietary switch from protein rich milk to carbohydrate rich chow, highlighting the involvement of a metabolic shift in maturation (81).The change in nutrient quality due to dietary transition at weaning triggers a switch from the nutrient sensing mTORC1 (Target of Rapamycin) pathway to the energy sensing AMPK (Adenosine Monophosphate-activated Protein Kinase) signaling, which in turn supports the establishment of the functionally mature beta cell phenotype (86). mTORC1 signaling is essential for DNA methylation dependent repression of disallowed genes and the developmental metabolic reprogramming underlying beta cell functional maturation (87). Beta cell specific loss of Raptor, a key subunit of mTORC1, was shown to cause reduced expression of Dnmt3a. The consequent hypomethylation and de-repression of disallowed genes (*Hk1*, *Dlk1*, *Pdgfra*, *Oat* and *Mylk*) and beta cell immaturity genes (*Dlk1* and *MafB*) led to impaired glucose stimulated insulin secretion (GSIS) and reduced beta cell mass due to beta cell-death (87). These data suggest that nutrient sensing pathways direct metabolic changes underlying beta cell maturation *via* DNA methylation patterning. The link between metabolic changes and DNA methylation is underscored by the dependence of DNA methyltransferase and DNA demethylases on metabolites such as S-adenosyl methionine (SAM) and a-ketoglutarate (a-KG), respectively (88), the levels of which depend on nutrient quality such as the extent of carbohydrate, protein, and fat intake, as well as flux through the TCA cycle. Altogether, changes in nutrient sensing and metabolic pathways during the neonatal growth phase orchestrate DNA methylation dependent changes in metabolism that define beta cell functional maturity.

The process of functional maturation also coincides with beta cells acquiring the capacity for compensatory expansion in response to increased insulin demand (81). The functionally mature beta cell phenotype is therefore not only GSIS competent, but also capable of adapting to metabolic challenges. Beta cell replication is a key mechanism that maintains beta cell mass in postnatal life by contributing to growth and adaptive expansion (71). Besides the regulation of genes involved in beta cell metabolism, DNA methylation also controls the expression of genes involved in proliferation (65). The capacity of beta cells to undergo adaptive proliferation declines with age (89, 90), and corresponds to increased GSIS due to age related changes in the methylome of the endocrine pancreas (65). Age-dependent changes in DNA methylation patterning are now well-recognized to reflect functional cellular aging (91, 92). Genome-wide methylome profiling of beta cells with age shows that promoters of genes encoding pro-proliferation proteins, such as Mki67, Cyclin D3, and Cyclin dependent kinases (Cdks), undergo *de novo* methylation. Age-associated *de novo* DNA methylation in islets has also been shown to loosen the epigenetic barrier between endocrine and acinar identities (93), and points to an epigenetic drift towards acinar epigenomic fate with age. On the other hand, distal regulatory regions of genes involved in the maintenance of beta cell identity and function such as *Pdx1*, *Nkx6.1*, *NeuroD1*, *Foxa2*, *Mnx1*, *Kcnj11*, *Abcc8*, and *Gck* become demethylated with age, corresponding to age-related improvement in GSIS (65). The distal regions that lose methylation with age, are typically marked with signatures of active enhancers in young mice, which likely primes them for future demethylation (65).

While the mechanisms that regulate age-associated DNA methylation changes are far from clear, Dnmt3a mRNA levels appear to be much higher in islets from older mice (6-26 months) compared to early neonatal life (1-2 weeks) (94). This is surprising, given the reduction of Dnmt3a during maturation. It is likely that Dnmt3a mRNA expression is downregulated upon maturation, and increases again in the older adult mice to support the *de novo* methylation and repression of cell-cycle genes. DNA methylation has been shown to intersect with polycomb protein dependent chromatin organization (95), and polycomb proteins play an integral part in age-dependent regulation of beta cell proliferation and function (96–98). It is, therefore, likely that the age-dependent changes in the proliferative and functional capacity of beta cells are co-regulated by an interplay of polycomb complexes and DNA methylation. In line with this, the distal regulatory regions that undergo age-dependent DNA demethylation in beta cells, are also marked by the loss of polycomb-dependent histone 3 lysine 27 trimethylation (H3K27me3) (65). Collectively, dynamic changes in the methylome lead to repression of the proliferative program and up-regulation of the beta cell functional programs with age in mice. Interestingly, while mouse and human beta cells follows the same trend for age-dependent changes in cell-cycle genes expression, the expression of beta cell function genes declines with age in humans, unlike in mouse beta cells (99). This may likely be a reflection of differences in physiological factors such as the body size and lifespan of the two species.

In addition to age, biological sex is another key variable that dictates DNA methylation patterns; in large part due to the fact that females display a DNA methylation dependent inactivation of the extra X-chromosome to regulate the dosage of X-linked gene in females (100). Several studies suggest that females secrete more insulin compared to males and are more insulin-sensitive (101, 102). A comparison of global DNA methylation patterns in human islets from males and females identified DNA methylation differences corresponding to 61 genes located on X-chromosomes and 18 autosomal loci, which also display sex-related differences in gene-expression (103). Three such genes, *NKAP*, *APLN* (both X-linked), and *SPESP1* (autosomal), that showed higher methylation and reduced mRNA in female islets, were found to regulate insulin secretion in functional assays. In addition, this study identified two miRNA loci that were differentially methylated and expressed between male and female islets, correlating with the observed sex differences in islet expression of multiple target genes for these miRNAs (103). These data point to the existence of sex-specific differences in human islet methylome that are highly relevant to the islet functional phenotype, and can partly explain the sex-specific differences in metabolic homeostasis.

**Figure 3:**
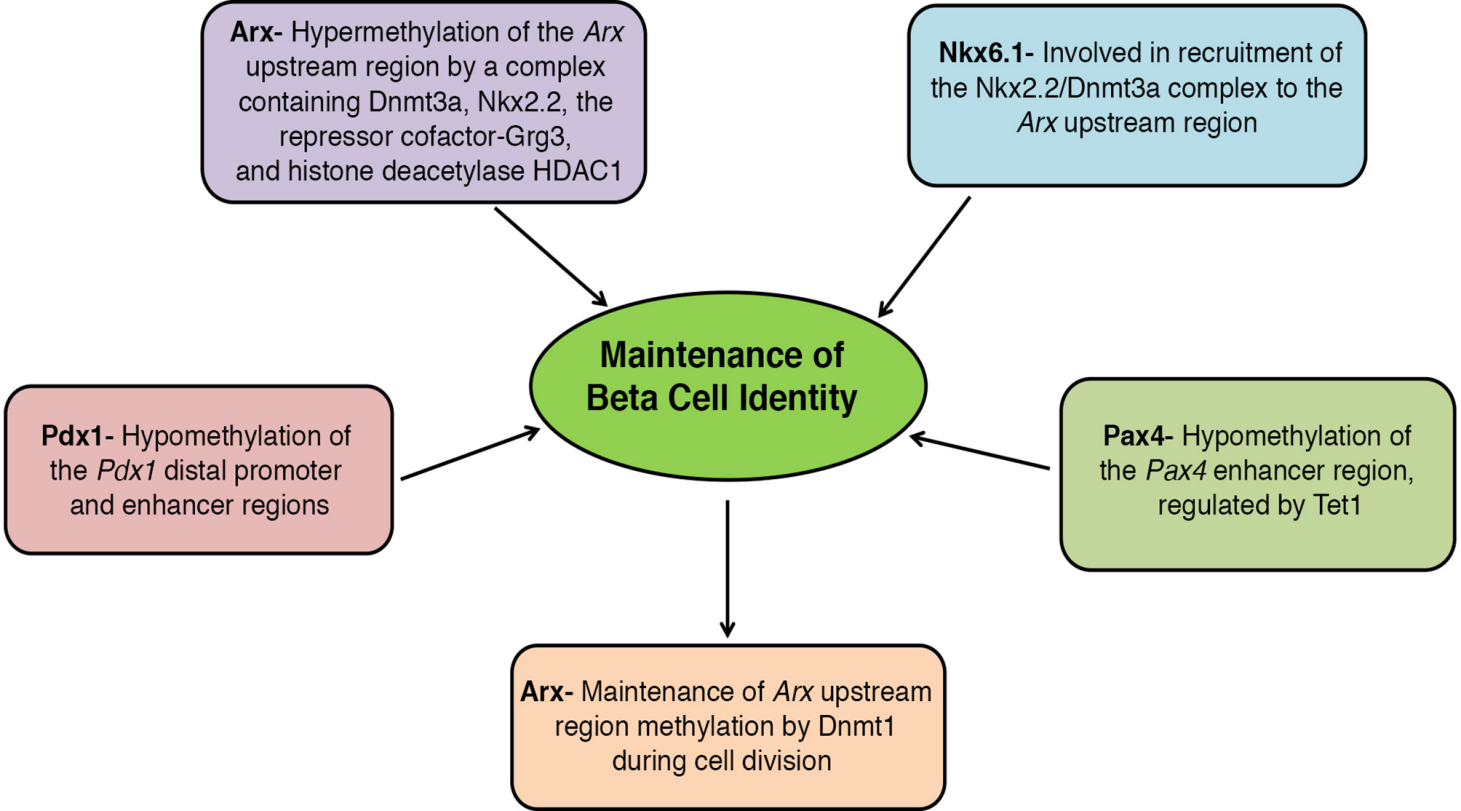
Key players involved in DNA methylation dependent regulation of beta cell identity: Hypermethylation of the alpha cell lineage regulator gene, *Arx*, represses its expression in beta cells, while hypomethylation of *Pdx1* and *Pax4* in beta cells is essential to maintain their expression. The specificity of DNA methylation patterning is ensured by the interaction of DNA methyltransferase Dnmt3a with transcription factors Nkx2.2 and Nkx6.1, allowing recruitment to specific sites. Once established, the beta cell specific DNA methylation patterns are maintained by the maintenance methyltransferase Dnmt1.

Replication of a terminally differentiated cell type (such as beta cells) presents a unique challenge for the cell to faithfully transmit the epigenetic patterns that define the differentiated phenotype, from the parent- to daughter-cells. The maintenance methyltransferase Dnmt1 serves to maintain beta cell identity during cell division by reestablishing the DNA methylation patterns required for the continued repression of alpha cell lineage, such as the methylation of *Arx* (64). Consequently, the inactivation of *Dnmt1* in beta cells leads to the de-repression of *Arx* and trans-differentiation of beta- into alpha-cells, causing a reduction in beta cell mass (64). The DNA methylation dependent repression of *Arx* in beta cells involves the recruitment of a complex containing the methyl-binding protein MeCP2 and histone methyltransferase Prmt6 to the methylated region of *Arx*, which establishes a repressive chromatin profile. In the absence of *Dnmt1*, loss of DNA methylation prevents restoration of the corresponding repressive chromatin state in the daughter cells (64). Notably, beta cell specific loss of *Dnmt1* does not result in a large-scale de-repression of silent genes; yet again pointing to the lineage specificity of epigenetic programs. Instead, Dnmt1 appears to maintain the barrier between developmentally related cell lineages, such as alpha- and beta-cells. The conversion of beta- to alpha-cells driven by the loss of *Dnmt1* is a slow process, due to the gradual dilution of DNA methylation marks with each round of cell division, and a refractory period before a beta cell can re-enter cell-cycle. Combined with the age-related decline of beta cells undergoing successive rounds of cell division, these factors amount to the observed slow rates of beta cell trans-differentiation. Of note, alpha cell specific loss of *Dnmt1* does not result in their trans-differentiation to beta cells, and requires the combined ablation of *Dnmt1* and *Arx* (104), suggesting that alpha cell identity maybe the default epigenetic endocrine state. This is noteworthy, as diabetes is associated with beta-to-alpha trans-differentiation, which occurs at a slow rate (105). A recent study suggests that inhibition of binding of transcription factor Foxo1 to the *Arx* promoter is essential for *Arx* hypermethylation to maintain beta cell identity (106), suggesting that transcription factors can regulate the maintenance of endocrine identity in a DNA methylation dependent manner (**Figure 3**).

In light of the above discussion, it is conceivable that changes in nutrition and metabolism at any point in postnatal life can alter the DNA methylation patterns underlying beta cell identity and maturity, which can in turn impact the functional response of beta cells. Such plasticity of the beta cell epigenome is essential for adaptation to changing insulin demands. However, altered nutrition during the sensitive growth phase in early life, or sustained high demand for insulin in adult life, can disrupt the epigenetic program and lead to an insufficiency of functional beta cell mass, due to poor growth or poor adaptation, respectively.

### Developmental Origins of Diabetes Risk: The Role of DNA Methylation

A large number of studies across multiple species point to a strong link between developmental perturbations such as poor intrauterine nutrition and fetal growth impairment, and diabetes development in later life, and suggest that early epigenetic events can contribute to disease risk and pathogenesis (14, 107–110). For example, studies on the Dutch Hunger Winter famine revealed that intrauterine under-nutrition and low birth weight predispose to an increased risk for developing diabetes in subsequent generations (111). This phenomenon, termed the “thrifty phenotype hypothesis”, proposes that under-nutrition and poor growth during fetal life can drive permanent changes in the glucose-insulin axis (112). On the other hand, maternal over-nutrition and gestational diabetes can also have an adverse impact on the metabolic fitness of the offspring (14). Epidemiological data show that exposure to maternal obesity or diabetes during fetal life predisposes the offspring to insulin resistance, impaired beta cell function, and high risk of diabetes (113, 114). Studies in rat models have shown that severe maternal diabetes leads to acute fetal hyperglycemia and consequent beta cell hyperplasia, as well as increased insulin output and inability to undergo adaptive beta-cell expansion in adult life (115, 116). Similarly, a recent study showed that maternal exposure to a Western style diet in non-human primates results in aberrant islet composition and insulin hypersecretion in the offspring (117). Collectively, these studies demonstrate that optimal nutrition during development is essential for future metabolic health of the offspring.

As discussed earlier, DNA methylation homeostasis directly coupled with cellular metabolism and nutrient quality. The availability of the Dnmt co-factor S-adenosyl methionine (SAM). Cellular levels of SAM are determined by biochemical reactions involving methyl-donor vitamins such as folate and vitamin B12, amino acids such as methionine, choline, taurine, as well as flux through the TCA cycle (118). Thus, altered levels of these micro-and macro-nutrients during the growth phase can impact the epigenome and organ development. Indeed, folate and vitamin B12 excess in pregnant mice leads to islet dysfunction and impaired beta cell mass in in the offspring (119, 120). Of note, S-adenosyl homocysteine (SAH), the by-product of SAM utilization during the methylation reaction, is an inhibitor of DNA methylation (121). Importantly, the levels of SAH are elevated in obesity (122, 123), and can contribute to aberrant DNA methylation in the offspring in response to maternal obesity. Therefore, nutritional imbalances during fetal or neonatal life can alter the epigenetic program responsible for the development, growth, and function of metabolic organs, thus predisposing the offspring to diabetes (14, 124). This is especially concerning, given the increasing popularity of vitamin supplements and energy drinks containing taurine. Environmentally induced epigenetic changes may also occur in the gametes and be transmitted to subsequent generations, thus contributing to the epigenetic inheritance of diabetes risk [reviewed in (125)] (126–128). In support of this, impaired glucose homeostasis in either parent has been shown to change the metabolism in the offspring, concomitant with specific epigenetic changes (111, 129, 130).

In line with the above discussion, intrauterine growth restriction (IUGR) leads to impaired pancreatic development, reduced functional beta cell mass, and higher risk for diabetes (131, 132). A large number of studies show that the DNA methylation signatures important for beta cell function and identity are disrupted in models of maternal malnutrition and IUGR, ultimately affecting beta cell homeostasis (14). Several studies have demonstrated that IUGR and maternal dietary variation alter the methylation patterns that govern the expression of transcription factors involved in beta cell identity and function, such as Hnf4a and Pdx1; ultimately leading to impaired functional beta cell mass and eventually diabetes (109, 133, 134). Genome-wide profiling of DNA methylation in islets from control and IUGR rats points to dysregulation of the islet methylome preceding diabetes onset. The genomic regions showing altered DNA methylation in IUGR islets include genes involved in beta cell proliferation, insulin secretion, islet vascularization, and cell death, correlating with changes in the corresponding transcripts and reduced functional beta cell mass (135). Similarly, overnutrition in mouse pups leads to islet dysfunction, corresponding with extensive alterations in the islet DNA methylome at weaning; many of these methylation changes occur at genes involved in islet function and control of endocrine identity (93).

Imprinted genes represent a key group of genes relevant to islet homeostasis and diabetes pathogenesis, that are developmentally regulated by DNA methylation (136, 137). These genes display differential allelic regulation of transcription based on parental origin. The DNA methylation patterns of imprinted genes are highly guarded during early embryonic development, to control the gene dosage of important regulators of growth and differentiation (138). Environmentally induced perturbations in the DNA methylation patterning of imprinted genes can therefore not only cause disease risk transmission, but also dictate the pattern of epigenetic inheritance. In line with this, disturbances in methylation of imprinted genes have been shown to relate to the inheritance of diabetes risk (139). For example, mutations in the imprinted locus *KCNQ1*, which encodes a potassium channel essential for beta cell function, have been shown to impart diabetes susceptibility when inherited maternally, due to altered DNA methylation in early development (136). Similarly, the methylation patterns of imprinted *DLK1*-*MEG3* locus are altered in islets from subjects with T2D, leading to repression of several maternally expressed miRNA. This in turn leads to upregulation of miRNA targets such as *IAPP* and *TP53INP1*, that are associated with beta cell dysfunction and cell death, thus contributing to T2D pathogenesis (137).The significance of imprinting control in islet homeostasis and metabolic health is further emphasized by imprinting disorders such as the Beckwith-Wiedemann Syndrome (BWS) and Transient Neonatal Diabetes Mellitus (TNDM) [reviewed in (11)]. In BWS, imprinting defects lead to loss of expression of cell-cycle inhibitor CDKN1C (p57^Kip2^), resulting in unchecked beta cell expansion and excessive beta cell mass, which causes hyperinsulinemia, and hypoglycemia (140). In TNDM, germline loss of maternal allele of the cell-cycle regulator *PLAGL1* results in its overexpression, which leads to transient beta cell dysfunction and plays a role in TNDM. Environmental changes, such as intrauterine hyperglycemia can also alter DNA methylation of imprinted genes to cause islet dysfunction and increase diabetes risk, as shown in mice for the *Igf2*/*H19* locus in islets (141).

These studies collectively show that DNA methylation patterns of several key regulators essential for the fully functional beta cell phenotype are dysregulated in response to adverse early developmental exposures. An abnormal nutritional milieu during fetal development and early growth may not only impede beta cell growth, but can also negatively impact the epigenetic program that dictates the mature beta cell phenotype. This can not only result in a deficit of functional beta cell mass, but also predispose the offspring to diabetes in adult life (14). Occurrence of such environmentally induced epigenetic defects in the beta cell program in the germline can potentially result in their transmission to subsequent generations, thereby presenting a mechanism for the epigenetic inheritance of beta cell dysfunction and diabetes risk (125).

### Dysregulation of Islet DNA Methylation Landscape in Diabetes

People with a family history of diabetes have a much higher risk of disease development (142–146). However, genetic linkage can account for only a small percentage of diabetes cases associated with family history. Furthermore, the incidence of both type 1- and type 2-diabetes (T1D and T2D) has increased over the past half-century at a rate that cannot be explained by genetic factors alone, and has been attributed to environmental factors such as altered nutrition and a sedentary lifestyle. Collectively, this suggests the involvement of epigenetic mechanisms that mediate the effect of environment on gene expression and cellular phenotype (145–148). As elaborated earlier, the involvement of epigenetic factors in diabetes risk is further underscored by studies on the effect of fetal environment (107–110), the critical role of epigenetic mechanisms such as DNA methylation in beta cell homeostasis (31, 64, 96, 97, 149, 150), and a direct link between metabolism and the epigenome (151–153). Changes in the cellular DNA methylation landscape, either due to single nucleotide polymorphisms (SNPs) that add or remove CpG sites, or in response to environmental factors, can alter the function of different metabolic tissues, including islets, and potentially contribute to diabetes risk and pathogenesis [reviewed in (9, 124, 154)].

Changes in DNA methylation patterns have been shown to directly contribute to beta cell defects in type 2 diabetes (T2D) (**Tables 1** and **2**). Several studies have demonstrated that promoters of genes important for beta cell identity and function such as *INS*, *PDX1*, *PPARGC1A* and *GLP1R* are hypermethylated in human islets from donors with T2D compared to islets from non-diabetic donors (134, 155, 156, 158), resulting in their decreased expression and the consequent impairment of beta cell identity and insulin secretion. In fact, such epigenetic changes can occur preceding T2D, as illustrated by a recent study in diabetic mice, that identified DNA methylation changes associated with differential expression of several genes. Notably, the differentially methylated and expressed genes were enriched in pathways associated with insulin secretion (161). Similarly, islets from obese mice show an upregulation of Dnmt3a, which leads to increased *Nkx6.1* promoter methylation, reduced Nkx6.1 and insulin expression, and consequently, impaired islet function (162).

**Table 1:** DNA methylation studies in islets from T2D subjects, techniques utilized, and the key findings.

Study comparing T2D versus non-diabetic islets	Technique utilized to study methylation status	Samples	Key finding
(155)	Bisulfite sequencing of the islet DNA	10 diabetic and 9 non-diabetic subjects	*PPARGC1* promoter is hypermethylated leading to its reduced expression in T2D islets
(156)	Sequenom’s MassARRAY Epi-TYPER protocol	9 diabetic and 48 non-diabetic subjects	*INS* expression is decreased in islets of T2D due to increased methylation of its promoter
(134)	Sequenom’s MassARRAY EpiTYPER protocol	9 diabetic and 55 non-diabetic subjects	Distal promoter and enhancer of *PDX1* is hypermethylated leading to its reduced expression in T2D islets
(157)	Infinium HumanMethylation27K BeadChip Assay	5 diabetic and 11 non-diabetic subjects	276 CpG loci affiliated to promoters of 254 genes showed differential DNA methylation in T2D islets
(158)	Sequenom’s MassARRAY EpiTYPER protocol	10 diabetic and 55 non-diabetic subjects	*GLP1R* expression is decreased in islets of T2D due to increased methylation of its promoter
(159)	Infinium HumanMethylation450K BeadChip Assay	15 diabetic and 34 non-diabetic subjects	1,649 CpG loci and 853 genes showed differential DNA methylation in T2D islets. 102 genes showed both differential DNA methylation and gene expression in T2D islets.
(160)	Whole-genome bisulfite sequencing	6 diabetic and 8 non-diabetic subjects	25,820 DMRs identified in islets from individuals with T2D

**Table 2:** DNA methylation status of the key genes related to beta cell homeostasis in human islets in the context of T2D.

Gene name	Gene function	Methylation status in T2D *vs* ND islets	References
*INS*	*G*lucose homeostasis	Hypermethylation	(156, 159)
*PDX1*	Insulin expression, beta cell identity	Hypermethylation	(156, 159, 160)
*PPARGC1A*	Insulin secretion	Hypermethylation	(155, 159)
*GLP1R*	Insulin secretion	Hypermethylation	(158)
*SLC2A2*	Glucose transporter	Hypermethylation	(160)
*EXOC3L2*	Beta cell exocytosis	Hypermethylation	(159)
*PER2*	Clock gene, metabolic homeostasis	Hypermethylation	(157)
*MADD*	Glucose-stimulated insulin secretion	Hypermethylation	(157)
*FTO*	Glucose-stimulated insulin secretion	Hypomethylation	(159)
*KCNQ1*	Insulin secretion, T2D risk linkage	Hypomethylation	(159)
*CDKN1A*	Proliferation and insulin secretion	Hypomethylation	(159)
*PDE7B*	Insulin secretion	Hypomethylation	(159)
*SEPT9*	Insulin secretion	Hypomethylation	(159)
*TCF7L2*	T2D risk linkage	Hypomethylation	(159, 160)
*ADCY5*	T2D risk linkage	Hypomethylation	(159, 160)
*NIBAN*	Survival, ER-stress	Hypomethylation	(157)
*BCL2*	Survival	Hypomethylation	(157)
*GUCA2B*	Insulin secretion	Hypomethylation	(157)
*CHAC1*	Survival. ER-stress	Hypomethylation	(157)
*NR4A1*	Survival	Hypomethylation	(157)
*CASP10*	Apoptosis	Hypomethylation	(157)
*SFRS2IP*	Pre-mRNA Splicing	Hypomethylation	(157)
*FOXA2*	Transcription factor	Hypomethylation	(157)
*SOX6*	Transcription factor	Hypomethylation	(157)
*PAX4*	Beta cell identity	Hypomethylation	(157)

Genome-wide profiling of changes in islet DNA methylation patterns in T2D using increasingly sophisticated methods has demonstrated that aberrant DNA methylation of transcriptional programs involved in beta cell function, survival and adaptation contributes to beta cell dysfunction in diabetes (**Table 1**). One of the first such studies identified 276 differentially methylated CpG sites related to promoters of 254 genes in islets from subjects with T2D, mapping to pathways involved in beta cell function, survival, and stress-response (157). Several of these differentially methylated regions (DMRs) translated in altered gene expression, with majority of them showing an inverse correlation, such that reduced methylation was coincident with increased expression and *vice versa*. Functional analysis of some of these candidates in human islets using RNAi based knockdown or exposure to stress stimuli underscored the relevance of these genes to dysregulation of beta cell homeostasis in T2D. These analyses, along with functional annotation, revealed epigenetic dysregulation of genes related to pathways such as transcriptional control (*FOXA2, PAX4, and SOX6*), ER-stress, function, and survival (*NIBAN*, *BCL2*, *PER2*, *GUCA2B*, *CHAC1*, *MADD*, *NR4A1*, *CASP10*, and *SFRS2IP*), in diabetic islets (157). A subsequent genome-wide DNA methylation profiling study using increased depth of mapping identified 853 genes displaying altered DNA methylation in T2D islets including key genes associated with T2D risk, namely *TCF7L2*, *FTO* and *KCNQ1*, highlighting the functional relevance of these DNA methylation changes. Similar to the prior study, several of the differentially methylated genes (*e.g. CDKN1A*, *PDE7B*, *SEPT9* and *EXOC3L2*) were differentially expressed (159), and relevant to islet function. More recently, whole-genome bisulfite sequencing has been used to generate an unbiased, single base resolution map of DNA methylation changes in diabetic islets. Such analysis led to the identification of 25,820 DMRs in islets from subjects with T2D, including regions known to regulate islet function, e.g., *PDX1*, *TCF7L2*, and *ADCY5* (160). Among the DMR associated genes, 457 candidates exhibited concomitant changes in gene expression, and included genes such as *SLC2A2*, *PDX1*, and *GLP1R* that regulate islet function. The DMRs in T2D islets were found to be enriched in binding sites for transcription factors relevant to beta cell function such as FOXA2, NEUROD1, MAFA, and PDX1 (160). This suggests that transcription factors regulate the human islet gene expression program in coordination with DNA methylation patterning.

Alterations in DNA methylation have also been shown to contribute to T1D pathogenesis (163). T1D is characterized by autoimmune destruction of the insulin producing beta cells, and both genetic as well as non-genetic factors contribute to T1D susceptibility (164–166). Several genes associated with T1D susceptibility have been identified, with ~50% of the T1D risk heritability contributed by the MHC locus alone (167), suggesting a major contribution of genetic factors. However, the high average concordance rate of T1D between monozygotic twins (~ 50%) and the rapid rate of increase in T1D incidence over the past few decades suggest the involvement of epigenetic factors. Several studies on blood samples from monozygotic twins discordant for T1D have demonstrated the relevance of altered DNA methylation in immune cells to T1D pathogenesis, with significant methylation changes at genes of immediate relevance to T1D pathogenesis such as *HLA* genes, *INS*, *IL-2RB*, *CD226*, *NFKB1A, TNF*, *INS-IGF2, CLEC16A*, and *GAD2* (163, 168, 169). While majority of the studies on DNA methylation differences in T1D have understandably focused on immune cells, a few studies have also demonstrated DNA methylation changes in islets in the context of autoimmune beta cell destruction in the NOD (non-obese diabetic) mice. For instance, one study on disease progression in the NOD mice showed that the inflammation leads to increased expression of Dnmt enzymes, resulting in altered DNA methylation at the *INS* locus and consequent reduction in insulin expression (170). Beta cell loss during T1D progression is accompanied by the release of unmethylated *INS* DNA in blood in humans (171–173), and similar release of unmethylated *Ins1* and *Amylin* DNA fragments in the sera of NOD mice (171, 174). Recent data shows that an inflammatory milieu can alter DNA methylation in human islets *via* TET2 dependent DNA demethylation, concomitant with increased expression of inflammatory and immune pathways genes (175). In line with this, emerging evidence suggests that Tet2 may regulate beta cell response to inflammation in T1D (176). Future studies in islets from auto-antibody positive, non-diabetic cases as well as T1D with residual beta cells in the pancreas may help determine if islet DNA methylation patterns are altered in human islet autoimmunity, and whether they directly contribute to beta cell dysfunction and cell-death in T1D.

While there is abundant evidence that the epigenetic programs that regulate functional beta cell mass are disrupted in diabetic islets, the extent of their causal relationship to disease pathogenesis is not completely clear. One can argue that the DNA methylation changes observed in T2D islets maybe secondary to hyperglycemia. However, the functional analysis of T2D associated DMRs, as well as the overlap of several of these DMRs with regions associated with T2D risk point to causality. Diabetes risk factors such as obesity, aging, and hyperglycemia alter the methylation patterns of T2D associated islet DMRs in non-diabetic subjects, suggesting that epigenetic dysregulation occurs prior to diabetes onset (159). This observation further supports the causal role for such epigenetic changes. While majority of the genetic variations associated with T2D have been shown to be related to islet function (177), they only amount to a minor fraction T2D heritability. It is likely that a combination of genetic and epigenetic factors contribute to diabetes risk and susceptibility, given that DNA methylation changes can be introduced due to genetic variations at even 1-2 CpG sites (178). This also appears to be the case for T1D, as demonstrated by a variant in the imprinted *DLK1*-*MEG3* locus on chromosome 14q32.2, which predisposes to paternally inherited T1D *via* altered imprinting control (179).

Loss of beta-cells in both T1D and T2D is preceded by progressive beta-cell failure due to impaired beta-cell identity, function, and survival. Functional defects in both scenarios involve beta cell de-differentiation and a recapitulation of the functionally immature phenotype. It is noteworthy that several of the T2D associated DMRs overlap with DMRs associated with endocrine differentiation of TET-deficient stem cells (55), suggesting that a dysregulated methylome may contributes to beta-cell de-differentiation. It is likely that some of these epigenetic changes initiate as an adaptation in response to increased islet workload, or, in case of T1D, to also evade immune assault. However, sustained environmental challenges such as cellular stress, inflammation, altered metabolism, and the inability to meet insulin demand can drive further dysregulation of the islet epigenome and lead to beta cell failure. Altered DNA methylation can also trigger DNA damage and make beta cells prone to apoptosis. This model is supported by studies showing that sustained oxidative stress or hyperglycemia drives a change in the islet transcriptional program from an adaptive mode to stress- response mode (180). In line with this, human islets exposed to lipotoxic stress, a T2D risk factor, exhibit reduced expression of DNMT3A and DNMT1, and increased expression of GADD45A, potentially explaining the mechanistic basis of CpG demethylation observed in T2D islets (159). Collectively, this discussion points to a causal role for islet DNA methylation dysregulation in diabetes risk and pathogenesis, and also underscore the combinatorial role of genetic and epigenetic factors in this process.

### Harnessing DNA Methylation for Diabetes Biomarkers and Therapies

Both type 1 and type 2 diabetes are complex disorders that impact multiple organs. A significant number of patients with diabetes develop serious health complications such as retinopathy, nephropathy, neuropathy, as well as cardiovascular disease (181). Loss of functional beta cell mass often begins long before disease diagnosis, and diabetes manifests after more than 50% beta cell mass is already lost (182). Therefore, early detection of beta cell defects may provide opportunities for disease prevention in people at higher risk, as well as inform the development of approaches for monitoring disease progression and personalized patient care. Currently, no established biomarkers exist for the assessment of beta cell loss preceding disease diagnosis. Detection of circulating cell-free DNA (cfDNA) has emerged as a non-invasive and practical tool for the development of blood-based biomarkers. CfDNA fragments are released into the blood due to cell-death, necrosis, or active secretion, and therefore potentially reflect disease related tissue changes. CfDNA is highly stable, and DNA methylation signatures of cfDNA fragments correspond to their tissue-of-origin (183, 184). Development of a comprehensive reference DNA methylation atlas of various human tissues now allows the robust and accurate determination of the tissue-of-origin of cfDNA in human plasma, in healthy and disease conditions (184). As discussed earlier, beta cell death is accompanied by the release of DNA fragments carrying beta cell specific methylation signatures in circulation, and can be detected by assaying for such signatures in the cfDNA. DNA methylation patterns of circulating cfDNA can, therefore, potentially serve as a biomarker to detect beta cell loss in diabetes.

Beta cell specific methylation patterns of human *INSULIN* (*INS*) gene have been widely used to detect beta cell death in the context of T1D (171–173, 185), and islet transplantation (172). The premise of these assays is that the circulating unmethylated *INS* promoter DNA fragments are exclusively of beta cell origin and indicate recent beta cell death (**Table 3**). Assays based on this principle have been used to assess beta cell death during T1D progression. A study focusing on a small cohort of at-risk subjects showed that the subjects who progressed to T1D displayed a modest increase in the circulating unmethylated *INS* DNA, corresponding to reduced insulin secretion in an oral glucose tolerance test (172). This study also reported that the half-life of circulating unmethylated *INS* DNA is only ~2 hrs, suggesting that such assays may only reflect acute beta cell death. Using this assay, this study showed that there is detectable beta cell death in the pre-diabetic period associated with reduced insulin secretion, followed by a dramatic increase in the peri-diagnosis period (172). A recent report described the development of a multiplex ultrasensitive assay that can detect as little as one beta cell genome equivalent. This assay was able to detect beta cell demise in several clinical contexts, such as in islet transplant recipients shortly after transplantation, and in patients with KATP congenital hyperinsulinism. However, unlike prior assays, this approach couldn’t detect an elevation of beta cell derived cfDNA in patients at risk for T1D (autoantibody-positive), and those with recent-onset or long-standing T1D (186). This could be due to several reasons. It is likely that beta cell death in T1D occurs in bursts, such that there may have been no ongoing cell death at the time of sampling. Reduced beta cell mass in pre- and early-T1D due to beta cell de-differentiation could also contribute to this. Longitudinal testing and development of additional markers is required to resolve this. Overall, while DNA methylation analysis of cfDNA holds a lot of promise, several variables can impact the outcome of such assays, including the rate of disease progression.

**Table 3:** Blood-based biomarker candidates T1D and T2D.

Genes	Context	Samples	References
*INS*	T1D	5 diabetic and 6 non-diabetic subjects	(171)
		10 T1D progressors and 10 non-progressors	(172)
Multiplex of *INS*, *INS* antisense, *LENG8*, *FBXL19*, *ZC3H3*, and *MTG1*	T1D	Multiple cohorts with total N=130 T1D (various ages of onset and stages of progression), N=32 autoantibody positive subjects, and N=97 controls.	(186)
*FTO*	T2D and Metabolic Syndrome	34 metabolic patients (25 with T2D and 9 with both MetS and T2D) and 11 control subjects	(187)
*TCF7L2*	T2D	93 diabetic and 93 non-diabetic subjects	(188)
*ABCG1*	T2D	2,770 participants, non-diabetic at baseline, followed prospectively, to detect progression toward T2D	(189)
		Follow-up of 25 372 participants; 1608 subjects of Indian descent and 7088 subjects of Europeans descent developed T2D	(190)
*PHOSPHO1*	T2D	2,770 participants, non-diabetic at baseline, followed prospectively, to detect progression toward T2D	(189)
		Follow-up of 25 372 participants; 1608 subjects of Indian descent and 7088 subjects of Europeans descent developed T2D	(190)
*TXNIP*	T2D	Independent prospective cohorts of Caucasian patients (N = 355, N = 167, and N = 645)	(191)
		Follow-up of 25 372 participants; 1608 subjects of Indian descent and 7088 subjects of Europeans descent developed T2D	(190)
	T1D	52 pairs of monozygotic twins, discordant for T1D	(192)
		32 EDIC (Epidemiology of Diabetes Interventions and Complications) Study participants as case group and 31 EDIC Study participants as control group	(193)
*ELOVL5*	T2D	11 pairs of T2D-discordant monozygotic twins	(194)

While biomarkers based on islet specific DNA methylation signatures in cfDNA have primarily focused on T1D, this approach may also be relevant to T2D (195, 196). Recent data demonstrate that the DNA methylation changes associated with T2D in beta cells and peripheral insulin sensitive tissues are reliably captured in the circulating DNA, supporting the suitability of cfDNA for developing T2D biomarkers (195, 196). Meta-analyses of multiple, highly powered genome-wide DNA methylation profiling studies comparing blood samples diabetic and non-diabetic subjects have identified several potential candidate loci, for which the T2D associated DNA methylation changes in blood are consistent across different ethnic populations (reviewed in (197). These include genes associated with T2D risk such as *FTO* and *TCF7L2* (187, 188), as well as genes involved in beta cell function and glucose homeostasis such as *ABCG1*, *PHOSPHO1*, and *TXNIP* (189–191). Notably, changes in *TXNIP* methylation in the peripheral blood have also been found to be associated with T1D and its complications (192, 193). In addition to identification of functionally relevant T2D associated DMRs such as those listed above, cross-comparison of DNA methylation profiling studies in multiple metabolic tissues can unravel T2D associated DMRs that are conserved across tissues. This is exemplified by the *ELOVL5*, which displays conserved T2D associated DMRs in islets and adipose tissue (194). Collectively, these functionally relevant candidates present as robust candidates for blood-based biomarkers for T2D (**Table 3**). Aging is an important “environmental” factor associated with increased risk of T2D, and leads to DNA methylation changes that dictate reduced beta cell replication and islet function with age (65). Genome-wide methylation profiling in non-diabetic donors (ages 26-74) showed that several age-related DNA methylation changes seen in human islets are conserved in blood samples, including some that associate with islet function and T2D. These data suggest that blood-based epigenetic biomarkers can be good predictors of islet function and T2D risk with age (196). DNA methylation patterns can also be leveraged to determine the differentiation status of stem cell derived products. For instance, promoter hypermethylation of disallowed genes has been used as marker of functional maturity during beta cell differentiation (198). As discussed earlier, the global levels of 5hmC progressively increase during the differentiation and maturation of beta cells. Therefore, in addition to locus specific DNA methylation patterns, global 5hmC content can also serve as a reliable index of beta cell differentiation and functional maturity (56).

Given the specificity and importance of DNA methylation patterning in beta cell homeostasis, and its dysregulation in diabetic islets, this epigenetic module has emerged as an attractive therapeutic target. Approaches that target DNA methylation have been successfully used for cancer therapeutics. For example, DNA methyltransferase inhibitors 5-aza-deoxycytidine (5azadC; Decitabine) and 5-aza-cytidine (5azaC; Azacitidine; Vidaza) are approved for use as anti-tumor agents (199). In a related approach, altering the availability of the methyl donor S-adenosyl methionine (SAM) has been proposed as an intervention in some clinical contexts such as depression, osteoarthritis, and liver disease (200). These agents, however, have a variety of documented harmful side-effects (199, 200). Vitamin B12 (folic acid), another methyl donor and a co-factor in SAM synthesis, is a widely used nutritional supplement, and has been effectively used to prevent neural tube defects (201). However, excessive folate intake during gestation and neonatal life can have obesogenic effects (202), and there are concerns regarding its potential oncogenic effects (201). Overall, the main challenge with all of these approaches is their generalized effect on DNA methylation, which can have deleterious off-target effects. While this currently precludes the use of these drugs for diabetes therapy, 5-aza-dC has recently been used *in vitro* to improve the efficiency of beta cell differentiation from human induced pluripotent stem cells (iPSCs) cells derived from subjects with T1D, which are otherwise known to differentiate poorly (203). DNA demethylation caused by 5-aza-dC treatment increases PDX1 expression during T1D-iPSC differentiation, thereby improving the differentiation efficiency (203). Thus, while global targeting of DNA methylation may likely find *in vitro* use for improving cell therapies, there is a need for developing highly specific targeting approaches that can restore the DNA methylation patterns that are disrupted in diabetes and promote a healthy islet phenotype.

Recent advances in genome editing using molecular tools such as CRISPR/Cas9 and TALEN now enable us to modify the DNA methylation patterns at specific regions in the genome, to either remedy disease specific epigenetic changes or introduce epigenetic patterns with potential therapeutic benefits. Locus specific epigenetic engineering has been used to target Dnmt or Tet enzymes to drive methylation or demethylation of specific genes (204). In a recent study, targeted DNA demethylation of the imprinted cell-cycle inhibitor gene *CDKN1C* was recently used to stimulate the expansion of adult human beta cells that typically do not replicate. Using a TALEN dependent targeting of TET1, Kaestner and colleagues simulated the DNA methylation patterns associated with beta cell hyper-proliferation in Beckwith Wiedemann Syndrome (BWS), where hypomethylation of the *CDKN1C* locus leads to reduced p57^Kip2^ and beta-cell hyper-proliferation. Using this approach, they were able to achieve the demethylation of *CDKN1C* locus and consequent reduction of p57^Kip2^ levels in human islets, and successfully induce replication of adult human beta cells (205). In another recent proof of principle study, CRISPR/Cas9 based targeting of Dnmt3a was used to drive the DNA methylation and repression of alpha cell-fate determinant gene *Arx* in mouse embryonic pancreatic progenitors, to promote beta cell lineage (63). Targeted epigenetic editing of developmentally regulated genes, therefore, has the potential to improve existing stem cell differentiation protocols by enhancing beta cell specification and functional maturation. Strategies involving the combinatorial, stage-specific epigenetic targeting of key genes that determine beta cell specification, functional maturation, and expansion, may soon become prevalent. Another potential approach in driving stage-specific epigenetic changes may be to mimic the environmental factors unique to a given developmental stage. It is increasingly being recognized that appropriate developmental niche is critical for the success of *in vitro* stem cell differentiation protocols (206). In this context, cellular disaggregation and reaggregation of immature beta cell clusters has been shown to promote DNA methylation changes associated with functional maturation (198). This process, intended to mirror the stage-specific changes in cell-cell interaction, is therefore able to induce physiologically relevant epigenetic changes. By combining environmental cues (*e.g.* nutrient availability) that simulate an *in vivo* scenario with epigenetic targeting and/or small molecule epigenetic modifiers, future work could dramatically improve the quality of stem cell derived islet products. Data generated from genome-wide DNA methylation profiling studies along with mechanistic work on the dysregulation of DNA methylation of key regulatory pathways (such as the imprinted *DLK1-MEG3* locus in T2D), is likely to inspire the development of novel therapeutic approaches for augmenting beta cell function and mass. Given the specificity of epigenetic engineering, such approaches may only have minimal off-target effects, and are thus likely to emerge as powerful therapeutic tools.

## Summary

The epigenome serves as a mechanism to adapt the behavior of a cell in response to changes in its environment, by interpreting the genome in a context specific manner and facilitating a transcriptional response accordingly. Methylation of the cytosine within the CpG residues in DNA is one of the most thoroughly studied epigenetic modifications, and one that can be faithfully inherited from parent to daughter cell during cell-division. DNA methylation is indispensable for shaping stage- and cell-type specific transcriptional programs, starting from early embryonic differentiation events to the maintenance of fully differentiated cellular phenotypes (9). While it is clear that the cellular environment shapes the methylome, the precise mechanisms underlying this plasticity are still not well understood. For instance, to what extent does 5hmC contribute to the demethylation of DNA versus serving as an independent regulatory module? What is the regulatory role, if any, of oxidized intermediates such 5fC and 5caC? Some of the work discussed in this review provides strong evidence that DNA methylation does not operate in isolation, but rather requires a coordinated regulatory interaction with other epigenetic modules (207, 208). Recent work, such as studies discussed in this review, also appears to suggest that cell-type and stage-specific transcription factors, including pioneer factors, play an important role in governing the spatio-temporal specificity of DNA methylation patterning. However, our knowledge of such coordinated interaction between cellular environment, signaling pathways, transcriptional programs, and multiple layers of epigenetic regulation is still in its infancy. Studies focusing on these aspects will provide a clearer understanding of the mechanistic underpinnings of gene-environment interaction, and identify sensitive temporal windows of methylome plasticity, genomic regions that are more- or less- vulnerable to methylation changes during a cell’s response to its environment, and the functional implications of such epigenomic flexibility.

The dynamic regulation and appropriate propagation of DNA methylation patterns is essential for the establishment and maintenance of functional beta cell mass, and accumulating evidence suggests that perturbations in the beta cell methylome are associated with beta cell pathologies and may contribute to disease development. Studies using mouse genetics, rodent models of metabolic disease, and stem-cell models of beta cell differentiation have been instrumental in shaping our current understanding of the critical role DNA methylation plays in the regulation of beta cell homeostasis. Environmental factors such as fetal growth impairment, altered nutrition, cellular-stress, aging etc. have been shown to predispose to diabetes, and can cause changes in DNA methylation patterns that regulate functional beta cell mass (14). Whether such changes in the methylome actively drive beta cell dysfunction, or merely reflect the metabolic dysregulation, or both, remains to be seen. While canonically DNA methylation has been viewed as a rather stable mark, emerging evidence shows that highly acute physiologically relevant changes in the methylome can and do occur in many tissue types (175, 209). While *in vitro* data suggests that acute changes in DNA methylation can occur in the islets (175), it is not yet clear whether similar changes occur *in vivo*. It is likely that different components of the cellular methylome respond differently to environmental stimuli, and such variations may depend on the genomic location. Whole-genome level mapping of DNA methylation patterns has provided a wealth of knowledge on the specific genes and pathways involved in the epigenetic control of beta cell homeostasis, during beta cell differentiation and in the context of diabetes pathogenesis. In addition, such data is likely to support the development of novel and accurate biomarkers for predicting diabetes risk and monitoring disease progression. The recent recognition of the plasticity of DNA methylation marks, combined with the advances in genome editing techniques, now offers the possibilities of targeted manipulation to remedy disease specific DNA methylation patterns or introduce patterns with therapeutic benefit (13). Future development of methods for cell specific delivery of epigenetic targeting modalities is likely to foster the development of highly precise therapeutic interventions for diabetes.

## Author Contributions

Both NP and SD contributed to the conception of this review, critical review of the literature, drafting and editing of the manuscript. All authors contributed to the article and approved the submitted version.

## Funding

Work in Dhawan laboratory is supported by grants from the National Institutes of Health (R01DK120523), the Wanek Family Project to Cure Type 1 Diabetes at City of Hope, and Human Islet Research Network (NIH) UC4 DK104162 (to SD).

## Conflict of Interest

The authors declare that the research was conducted in the absence of any commercial or financial relationships that could be construed as a potential conflict of interest.
